# Successful treatment with the mTOR inhibitor everolimus in a patient with Perivascular epithelioid cell tumor

**DOI:** 10.1186/1477-7819-10-181

**Published:** 2012-09-03

**Authors:** Constantine Gennatas, Vasiliki Michalaki, Paraskevi Vasilatou Kairi, Agathi Kondi-Paphiti, Dionysios Voros

**Affiliations:** 1 Oncology Clinic Second Department of Surgery, Aretaieion Hospital, Athens Medical School, Athens, Greece; 2 Pathology Department, Areteion Hospital, Athens Medical School, Athens, Greece; 3 Second Department of Surgery, Areteion Hospital, Athens Medical School, Athens, Greece; 4 Oncology Clinic Second Department of Surgery, Athens Medical School, Athens, Greece Areteion Hospital, 76 V.Sofias av, 115 28, Athens, Greece

**Keywords:** mTOR inhibitors, Metastatic perivascular epithelioid cell tumors

## Abstract

Perivascular epithelioid cell tumor (PEComa) is an extremely rare neoplasm that appears to arise most commonly at visceral (especially gastrointestinal and uterine), retroperitoneal, and abdominopelvic sites. Malignant PEComas exist but are very rare. These tumors represent a family of mesenchymal neoplasms, mechanistically linked through activation of the mTOR signaling pathway. Metastatic PEComa is a rare form of sarcoma for which no effective therapy has been described previously and that has a uniformly fatal outcome. Although there is no known effective therapy, the molecular pathophysiology of aberrant mTOR signaling provides a scientific rationale to target this pathway therapeutically. The difficulty in determining optimal therapy, owing to the sparse literature available, led us to present this case. On this basis, we report a case of metastatic retroperitoneal PEComa treated with an oral mTOR inhibitor, with everolimus achieving significant clinical response.

## Background

The PEComa family of tumors consists of related mesenchymal neoplasms that exhibit myomelanocytic differentiation and share a distinctive cell type, the perivascular epithelioid cell, or PEC [1,2].

The major members of this family include lymphangioleiomyomatosis (LAM), a disease predominantly presenting as numerous nodular and interstitial pulmonary lesions in premenopausal women; angiomyolipoma, commonly identified as an asymptomatic renal lesion with evidence of vascular, muscular and adipocytic differentiation; and PEComa, an epithelioid malignancy with clear-to-granular eosinophilic cytoplasm typically arising in the gastrointestinal tract, retroperitoneum, uterus or somatic soft tissues, composed of nests and sheets of epithelioid or occasionally spindled cells, intimately related to blood vessel walls [3,4].

Tumors of the PEComa family are rare and usually occur sporadically. LAM and angiomyolipoma also are seen at high frequency in patients with tuberous sclerosis complex (TSC), a disorder caused by mutation of TSC1 or TSC2, for which the gene products negatively regulate mTORC1 through inhibition of the mTOR kinase activator, RHEB [5]. Both TSC1 and TSC2 gene products are involved in multiple cellular pathways, including regulation of cell proliferation, migration and differentiation through inhibition of the Rheb/mTOR/p70S6 kinase-signaling pathway [6]. Inactivation of the tuberin/hamartin complex in TSC thus leads to the activation of mTOR and the phosphorylation of p70S6K and ribosomal protein S6, and further promotes translational initiation and cell growth. This mTOR pathway is reported to be inappropriately up regulated not only in TSC-associated AML, but also in sporadic angiomyolipoma or PEComas.

Most PEComas are benign tumors and do not recur after complete surgical resection. However, a subset of PEComas exhibits malignant behavior, with either locally invasive recurrences or development of distant metastases, most commonly in the lung. No effective therapy for malignant PEComa has been described. Recently, Italiano *et al.* described transient improvement in two patients with malignant PEComa treated with temsirolimus, an inhibitor of mTOR [7]. Additionally, Bissler *et al.*[8] have reported promising results from the use of the mTOR inhibitor sirolimus on renal angiomyolipoma and on LAM associated with the TSC. Subependymal giant cell astrocytoma (SEGA), another TSC associated neoplasm, can also be successfully managed by mTOR inhibition, and everolimus is already an FDA-approved drug for non-resectable SEGAs [9].

Altogether, these findings support the inhibition of mTOR as a rational therapeutic target in tumors occurring in patients with TSC as well as PEComas. On this basis, we report a case of metastatic retroperitoneal PEComa treated with an oral mTOR inhibitor, everolimus.

## Case presentation

A 63-year-old woman underwent resection of a retroperitoneal angiomyolipoma in 2009. A metastatic workup including a thoracic, abdominal and pelvic computed tomography (CT) 1 year later revealed retroperitoneal recurrence, which was completely resected. The surgical specimen was routinely processed for histology. The tumor was composed predominantly of spindle-shaped cells arranged in fascicles. These spindled cells, with eosinophilic cytoplasm, resembled smooth muscle cells.

The comparison with the tumor resected in 2009 described as angiomyolipoma showed the same morphology as the recurrent tumor. Immunohistochemistry showed the neoplastic cells positive for HMB 45, Melan-A, vimentin and CD 117 (focally). The neoplastic cells were negative for SMA, Pan CK, CD 34 and S-100.The pathological characteristics in combination with immunophenotypes are consistent with the diagnosis of PEComa.

**Figure 1:**
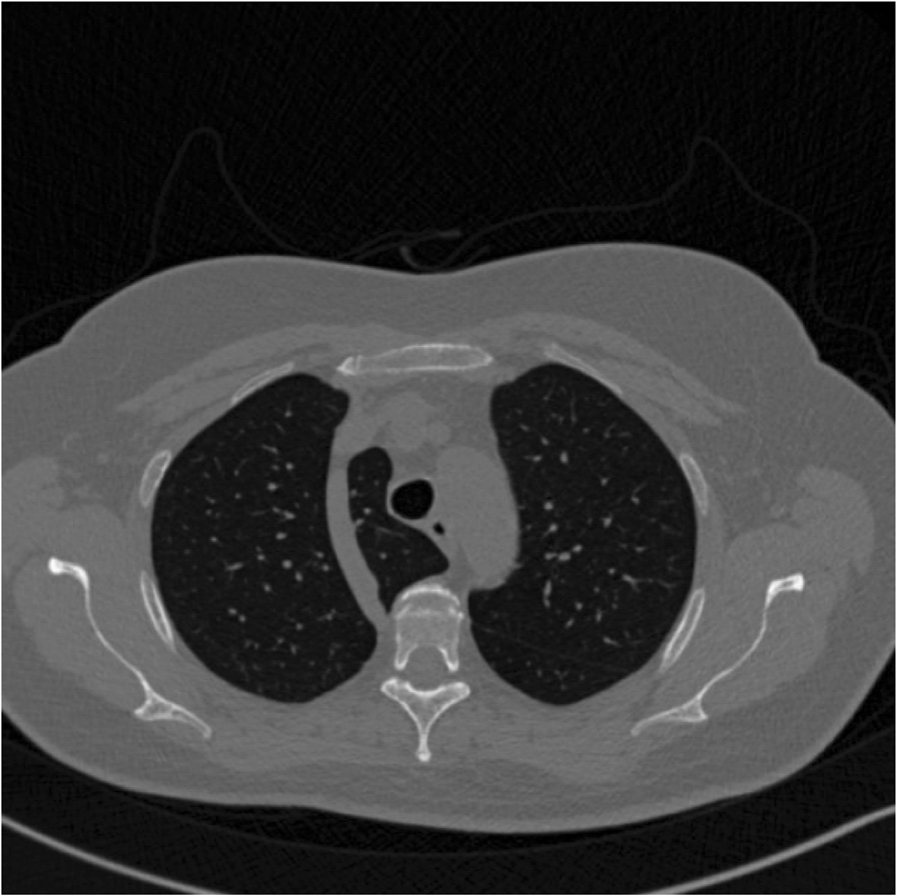
**Postoperative chest CT scan.** A chest CT scan showing diffuse, small, thin-walled cystic lesions in the parenchyma.

**Figure 2:**
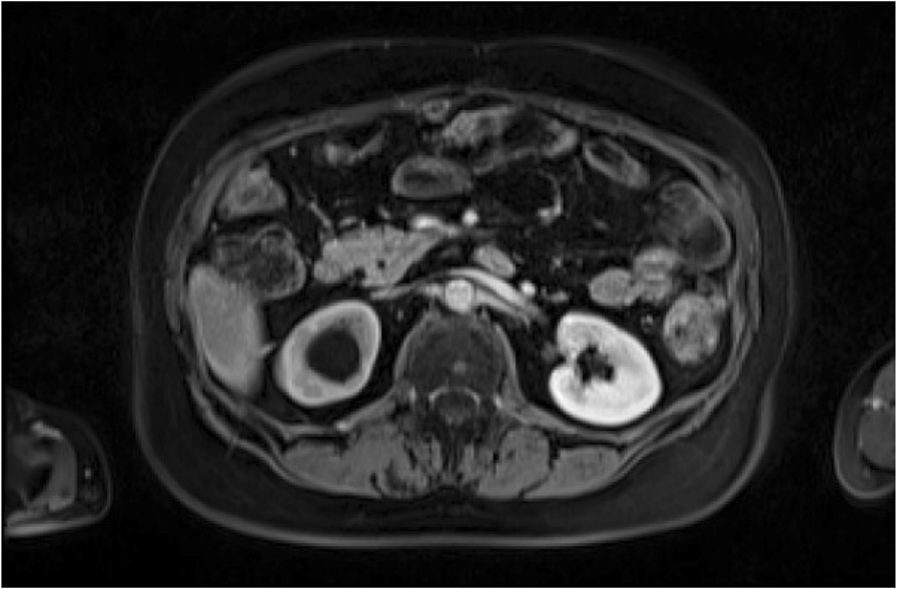
Postoperative abdomen scan before everolimus treatment.

The patient had remained disease free for 4 months when the follow-up CT scans revealed new lesions in the lungs and recurrence in the abdomen (Figures 1 and 2).

**Figure 3:**
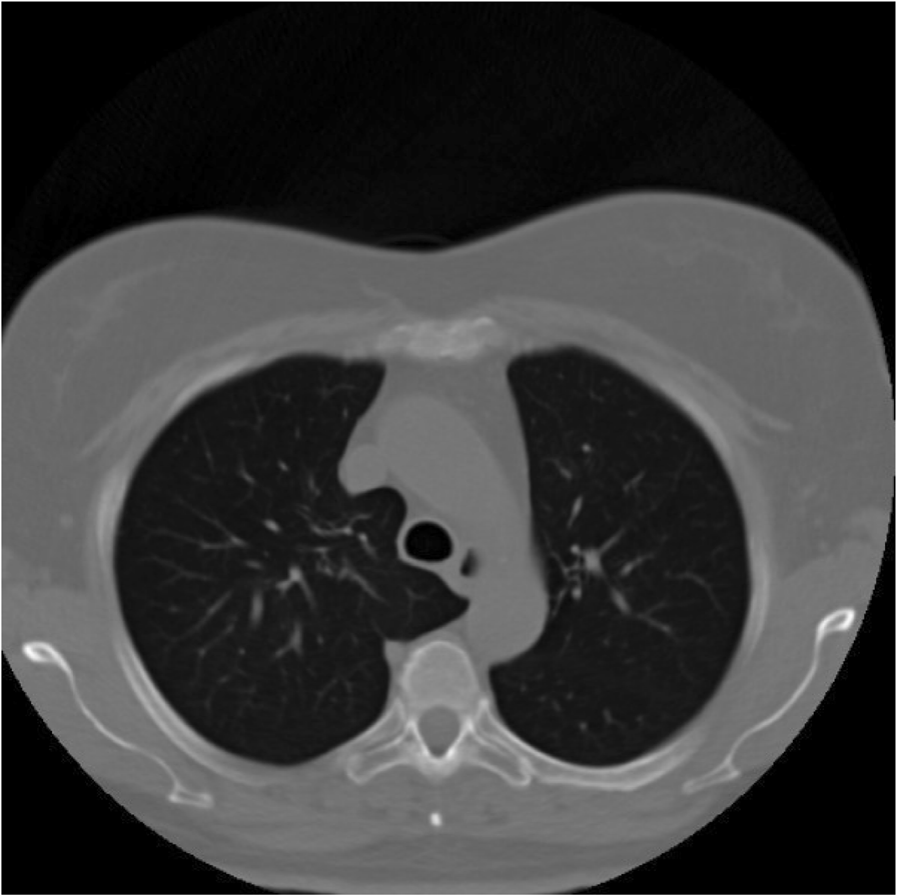
Chest CT scan after treatment with everolimus.

**Figure 4:**
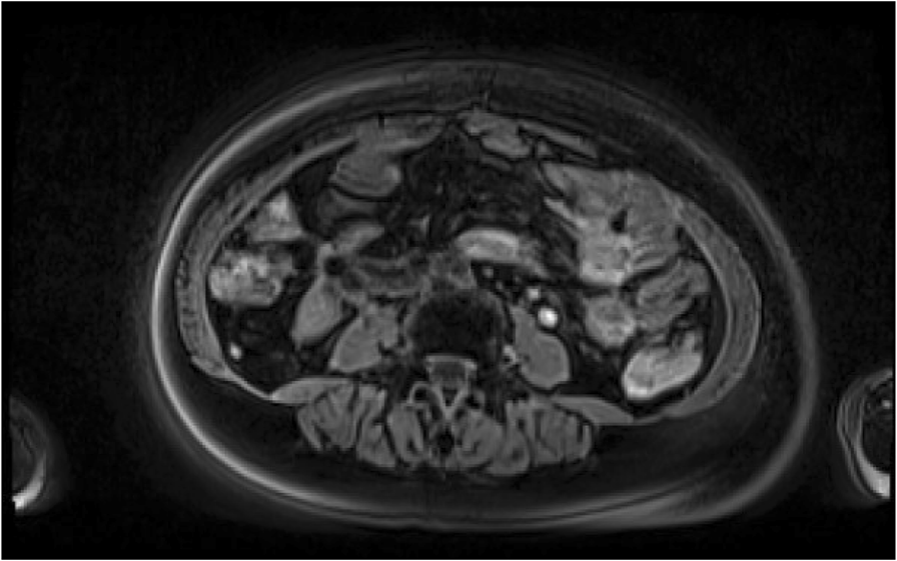
Abdominal scan after treatment with everolimus.

Surgery was initially considered, but the curability was estimated to be quite low considering the presence of lung metastases. Based on the immunohistochemical findings, which showed tumor cells positive for CD 117, she was placed on targeted therapy with the tyrosine kinase inhibitor imatinib mesylate. Treatment with imatinib was stopped with evidence of disease progression after 5 months. On the basis of published studies that showed activation of the mTOR pathway in PEComas, the institutional board approved everolimus as an off-label treatment for this patient [10]. This treatment option was discussed with the patient, who provided informed consent for treatment with everolimus as well as retrospective review of the medical records and evaluation of archival tumor specimens according to institutional review board–approved protocols. Everolimus was initiated at 10 mg per day. The dose was similar to the approved dosage in renal cell cancer. The first tumor evaluation carried out after 12 weeks revealed disappearance of the lung lesions and a MRI of the abdomen the same day revealed a significant response of the abdominal mass (Figures 3 and 4). Everolimus treatment was well tolerated, with grade 1 stomatitis and limited skin toxicity. The patient has continued with this treatment on an outpatient basis without signs of disease progression over 10 months before evidence of new recurrence in the retroperitoneum. She subsequently had an additional surgical resection and remains alive with the disease 37 months after primary diagnosis.

## Discussion

A subset of PEComas exhibits malignant behavior, with either locally invasive recurrences or development of distant metastases, most commonly in the lung. A challenge regarding these tumors is their management. Surgery seems to be the only approach for aggressive cases, as chemotherapy and radiotherapy have not shown significant results. However, this is based on few cases as no therapeutic trial has so far been implemented. There are obvious difficulties to performing a therapeutic trial mainly because of the rarity of the disease. Recent studies demonstrated TSC1/2 inactivation and m-TOR hyperactivation in non-TSC AMLs and in extrarenal PEComas using immunohistochemistry and Western blot analysis [11]. Based on the fact that PEComas share activation of the mTOR pathway with LAM and angiomyolipoma in many instances, we treated our patient with everolimus, an inhibitor of mTOR. We have observed significant clinical response with a near complete response of greater than 10 months’ duration.

Our data are consistent with findings published to date on the activity of mTOR inhibitors in tumors known to be biologically related to PEComas, specifically angiomyolipoma and LAM. After case reports of patients with renal angiomyolipoma and LAM responding to sirolimus were published, Bissler *et al.*[8] reported on the treatment of 25 patients with angiomyolipoma or LAM with sirolimus for 12 months followed by 12 months of observation. After 12 months of therapy, the angiomyolipoma volume decreased 53% but returned to 86% of baseline after the year of observation, which indicated the need for continued inhibition to maintain tumor shrinkage. Less impressive improvements in respiratory function were observed in patients with LAM, which also reversed on observation alone. Interestingly, facial angiofibromas associated with tuberous sclerosis also have significantly improved with sirolimus therapy in a case report [10].

Other evidence to support activation of the mTOR pathway in PEComas has also recently been described. Kenerson *et al.* reported immunohistochemical evidence of mTORC1 activity in 15 PEComas and absence of AKT phosphorylation in 14 tumors, which suggests the loss of TSC1 or TSC2 as potential mechanisms [11].

Similarly, Pan *et al.* described elevated phospho-p70S6K and reduced phospho-AKT in 11 of 12 PEComas. Seven of these tumors had loss of heterozygosity of the TSC2 region, and one additionally showed loss of heterozygosity of TSC1 [12].

The efficacy of mTOR inhibitors has also been explored in patients with a heterogeneous mix of other metastatic sarcomas, in each case with only a modest response rate. However, the status of mTOR activation of these sarcomas is unknown, although in one study the presence of S6 phosphorylation correlated with a higher likelihood of disease control with an mTOR inhibitor [9]. Taken together, these observations suggest that activation of mTOR through loss of the TSC1/TSC2 repressor complex, or potentially by other means, is likely a common and critically pathogenic event in PEComas.

Inhibition of mTOR has resulted in significant clinical activity in patients with PEComa and merits additional investigation in a prospective study. Absence of immunohistochemical evidence of TSC2 expression or the less specific presence of S6 phosphorylation may be predictive markers for responsiveness to inhibitors of mTORC1. These findings additionally unify the concept of PEComa, AML and LAM as closely related pathologic entities, from histology to genetic changes, to demonstrate the therapeutic benefit of mTOR blockade. Additionally, everolimus, which could be orally administrated, may be more convenient than infused drugs such as temsirolimus for both patients and medical facilities.

## Conclusion

Our report indicates that prolonged treatment of malignant PEComas with everolimus may result in durable tumor responses. Because of the very low frequency of these tumors and their aggressive nature, it may be difficult to conduct clinical trials; therefore, as indicated by our case and past reports, mTOR inhibitors may be one of the best treatment options for this malignant disease.

## Competing interests

The authors declare that they have no competing interests.

## Authors’ contributions

Conception and design: VM and DV. Provision of study material: CG, EKV, AKP. Collection and assembly of data: VM. Manuscript writing: VM. All authors read and approved the final manuscript.
